# Author Correction: Presynaptic endoplasmic reticulum regulates short-term plasticity in hippocampal synapses

**DOI:** 10.1038/s42003-021-02519-x

**Published:** 2021-08-10

**Authors:** Nishant Singh, Thomas Bartol, Herbert Levine, Terrence Sejnowski, Suhita Nadkarni

**Affiliations:** 1 Division of Biology, Indian Institute of Science Education and Research, Pune, Maharashtra India; 2 Computational Neurobiology Laboratory, Salk Institute for Biological Studies, La Jolla, CA USA; 3 Center for Theoretical and Biological Physics, Rice University, Houston, TX USA

**Keywords:** Computational neuroscience, Synaptic plasticity, Computational neuroscience, Synaptic plasticity

Correction to: *Communications Biology* 10.1038/s42003-021-01761-7, published online 23 February 2021.

In the original version of the Article, the Acknowledgements inadvertently omitted grant details and read “This work was supported by Welcome-DBT grant and IISER Pune”, which was corrected to “This work was supported by the Wellcome Trust/DBT India Alliance Grant IA/I/12/1/500529, the DST/INSPIRE Fellowship /2015/IF150919 and the Indian Institute of Science Education and Research Pune.” This has now been updated in the PDF and HTML versions of the Article.

