# Supplementary material for: Presynaptic endoplasmic reticulum regulates short-term plasticity in hippocampal synapses
Source: Commun Biol. 2021 Feb 23;4:241. doi: 10.1038/s42003-021-01761-7 (PMC7902852; doi:10.1038/s42003-021-01761-7)
Supplement: Supplementary file 2 — Supplementary Information [file 42003_2021_1761_MOESM2_ESM.pdf]

Presynaptic endoplasmic reticulum regulates short-term plasticity in hippocampal synapses

Nishant Singh[1], Thomas Bartol[2], Herbert Levine[3], Terrence Sejnowski[2], and Suhita Nadkarni[1,*]

[1]Division of Biology, Indian Institute of Science Education and Research, Dr. Homi Bhabha Road, Pune, India
[2]Computational Neurobiology Laboratory, Salk Institute for Biological Studies, 10010 N Torrey Pines Rd, La Jolla, CA 92037, USA
[3]Center for Theoretical and Biological Physics, Rice University, Houston, TX 77005-1827, USA
[*]Correspondence: suhita@iiserpune.ac.in

Supplementary Information

Supplementary Note 1

fig. 5a: $f(x) = sqrt((1-x)/(n*x))$ for CV; fitting parameters: n

fig. 5a: $f(x) = a - exp(b*(x+c))$ for Pr_2; fitting parameters: a,b,c

fig. 5g: $f(x) = a/x + b$; fitting parameters: a,b,c

fig. 6a: $f(x) = (1-(1-x)^{(a*x^b)})/x$; fitting parameters: a,b[1]

fig. 6b: $f(x) = a + exp(b*x+c)$; fitting parameters: a,b,c

fig. 6c: $f(x) = a - exp(b*(x+c))$; fitting parameters: a,b,c

fig. 6d: $f(x) = a + 14*(1-exp(b*x+c))$; fitting parameters: a,b,c

Supplementary fig. 2b1-2: $f(x) = 1/(a+exp(b*x+c))$; fitting parameters: a,b,c

Supplementary fig. 2c1-2: $f(x) = (1-(1-x)^{(a*x^b)})/x$; fitting parameters: a,b[1]

Supplementary fig. 2h1-2: $f(x) = a + exp(b*x+c)$; fitting parameters: a,b,c

Supplementary fig. 9a1-2,b1-2: $f(x) = a*x^b*exp(-c*x)$; fitting parameters: a,b,c

Supplementary Figure 1: Prevalence of axonal smooth ER.

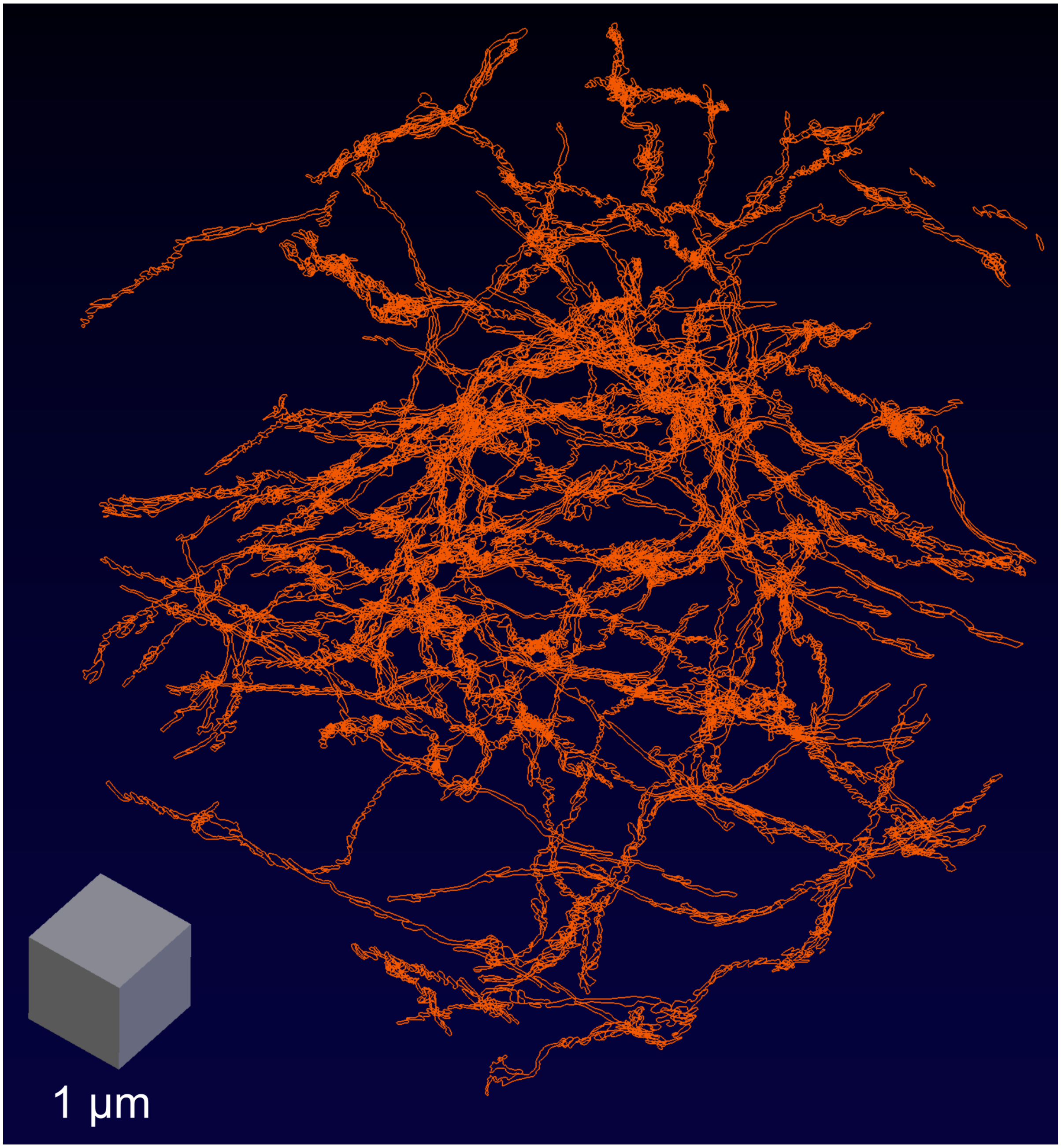

Figure 1. The hand segmented contours of smooth ER seen in subsample of 69 of these axons are shown highlighted in orange. Smooth ER in axons of stratum radiatum of hippocampal area CA1 was observed in all of the 449 axons identified in a dense serial section electron microscopic 3D reconstruction. The scale cube is 1 micron on a side.

Supplementary Figure 2: Synchronous and asynchronous vesicle release in paired pulse protocol.

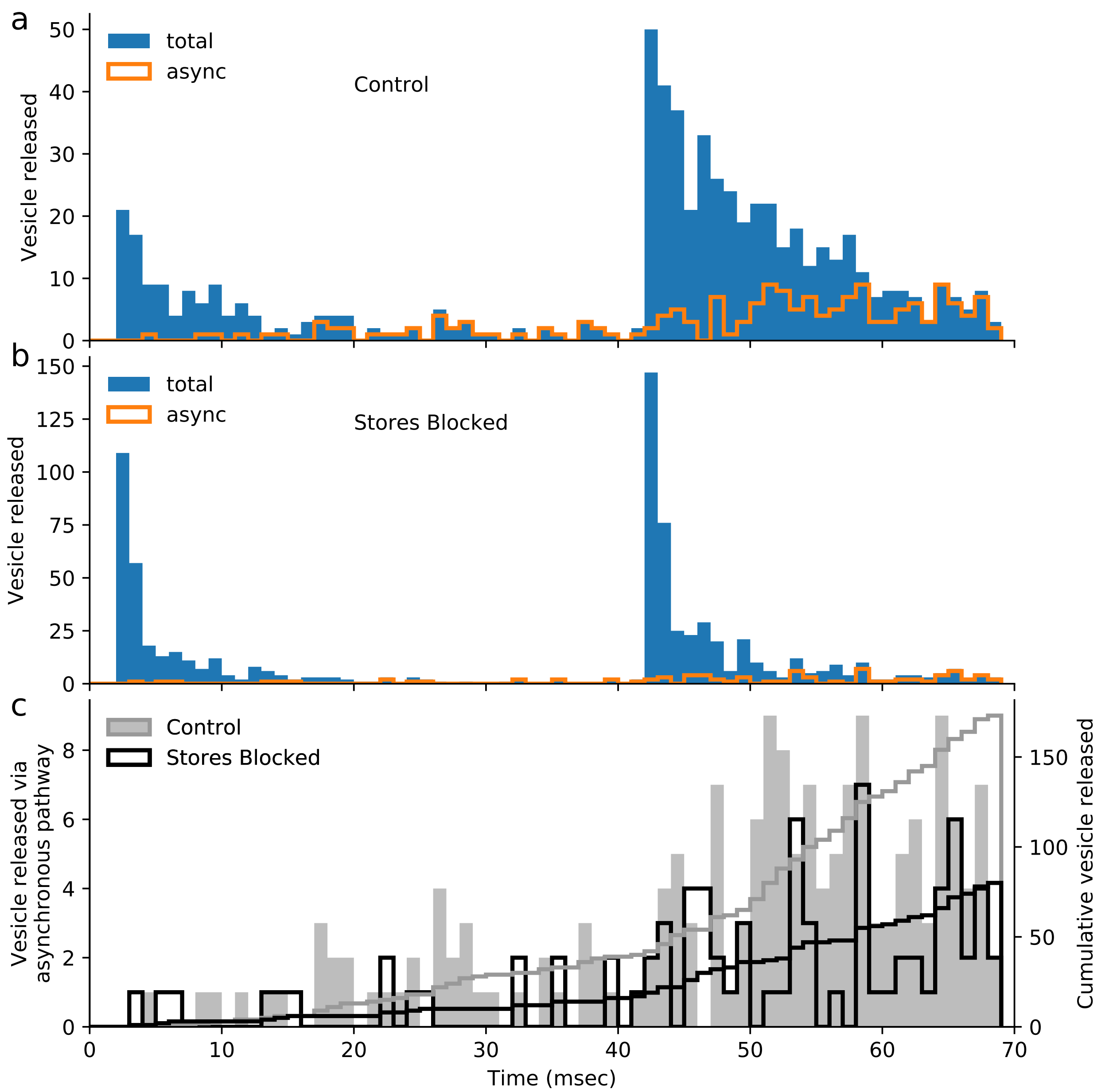

Figure 2. **(a)** Total vesicles released in 2000 trials of Control synapse. **(b)** Total vesicles released in 2000 trials of Stores blocked synapse. **(c)** Comparison of asynchronous release between the two synaptic configurations.

Supplementary Figure 3: ER contribution to paired-pulse ratio for 20ms ISI.

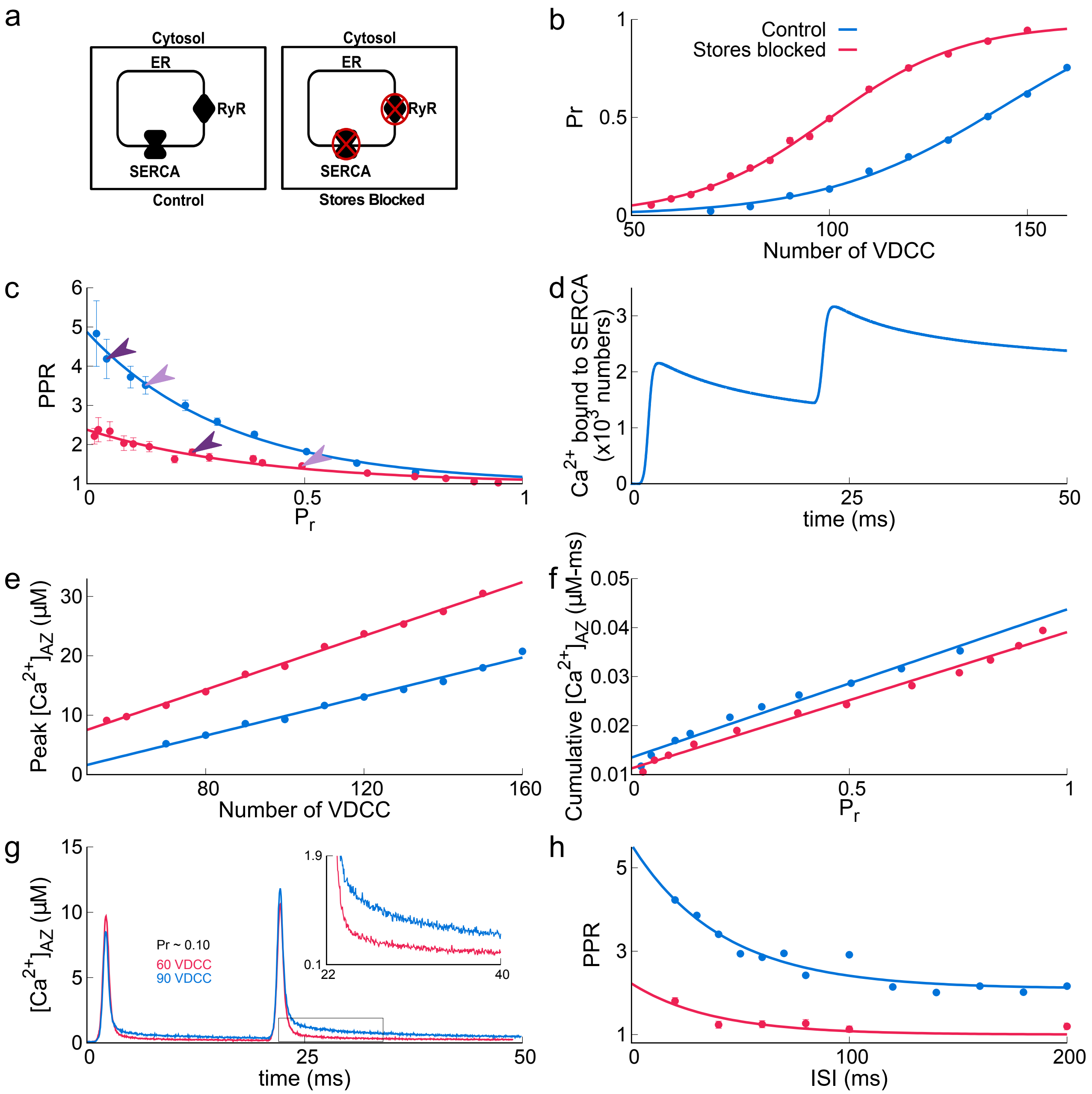

Figure 3. **(a)** Simulation is performed in canonical synapse for two model setups—'Control': Ryanodine receptors and SERCA pumps are present on the ER; 'Stores Blocked': SERCA pumps are blocked, therefore, no calcium ion is present in ER and RyR is redundant. **(b)** Variation of release probability of a vesicle (Pr) with number of VDCCs. **(c)** Inverse relation of paired-pulse ratio (PPR) and intrinsic Pr for various synaptic configurations. Dark purple arrows indicate the PPR corresponding to 80 VDCCs and light purple correspond to 100 VDCCs for canonical synapse. **(d)** Amount of Ca^{2+} ions bound to SERCA in response to a paired-pulse. **(e)** Variation in peak calcium concentration at the active zone, $[Ca^{2+}]_{AZ}$, with the number of VDCCs. **(f)** Cumulative calcium concentrations over 20 ms at the active zone in response to the second AP in a paired-pulse protocol for various Pr. **(g)** Calcium concentration at the active zone, $[Ca^{2+}]_{AZ}$ for Pr = 0.1. The colored text describes the corresponding number of VDCCs in each of the configurations to arrive at Pr = 0.1. Inset: Box area zoomed-in to show details of base level $[Ca^{2+}]_{AZ}$ concentration after the second AP. **(h)** Variation of paired-pulse ratio for different ISI. Data are mean ± s.d.

Supplementary Figure 4: Paired-pulse ratio (PPR) and intrinsic release probability (Pr) are insensitive to changes in SERCA density and calcium binding rates.

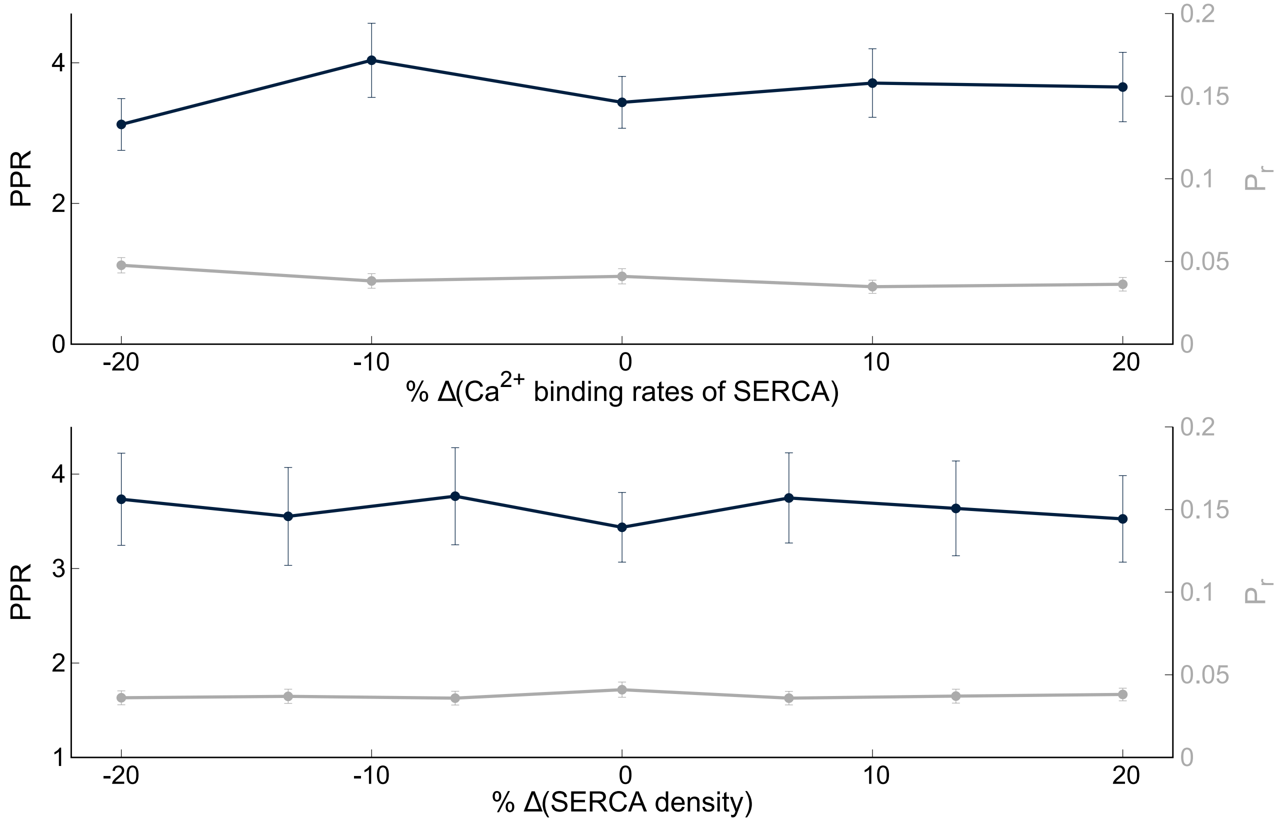

Figure 4. **(a)** PPR (black, left 'y' axis) and Pr (grey, left 'y' axis) for variation in calcium binding rates of SERCA. **(b)** PPR (black, left 'y' axis) and Pr grey, left 'y' axis) for variation in SERCA pump density. Data are mean ± s.d.

Supplementary Figure 5: Minor contribution of RyR to paired-pulse ratio.

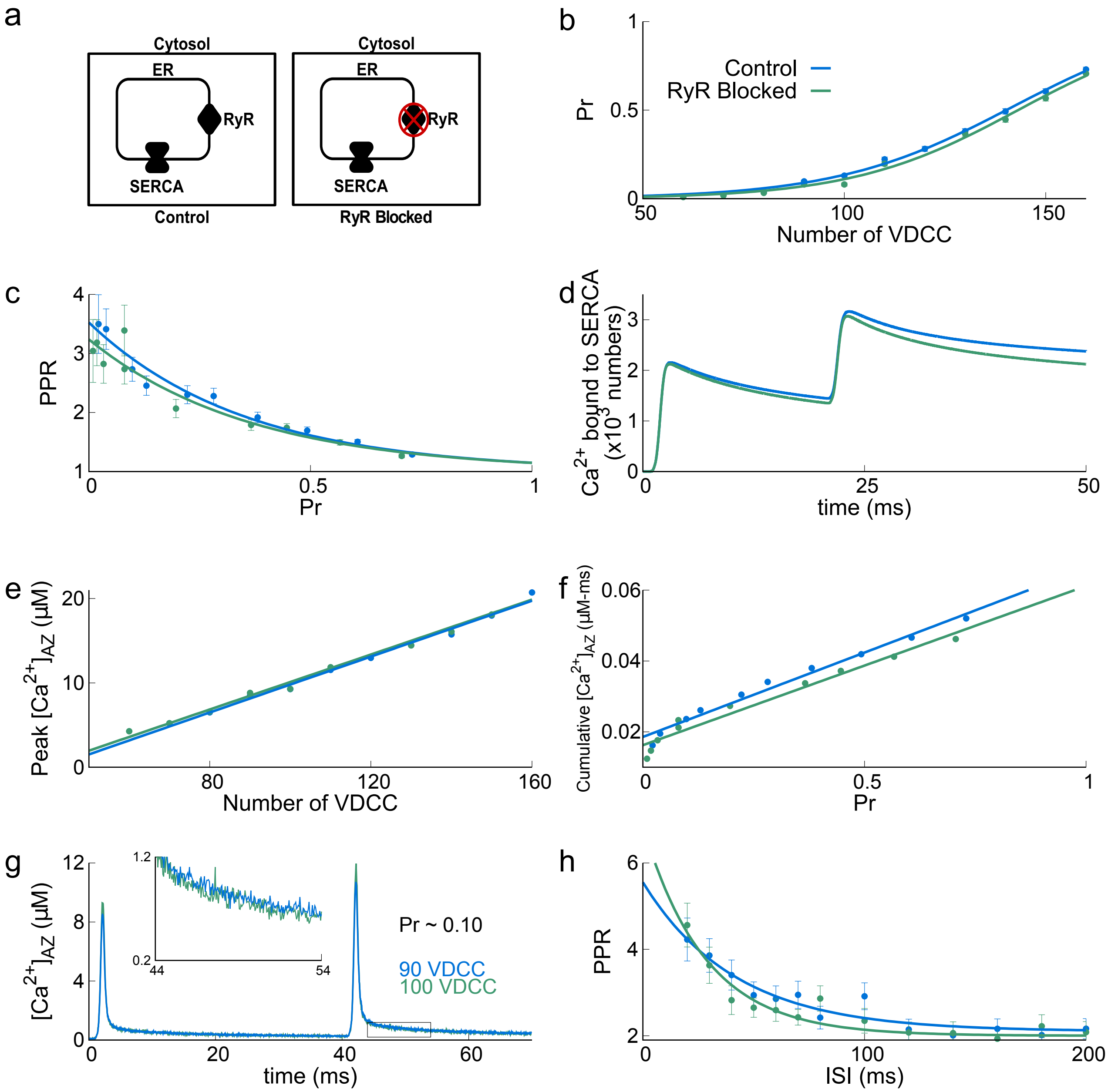

Figure 5. **(a)** Simulation is performed in canonical synapse for two model setups—'Control': Ryanodine receptors and SERCA pumps are present on the ER; 'RyR Blocked': SERCA pumps are present on ER and RyR is blocked. **(b)** Variation of release probability of a vesicle (Pr) with number of VDCCs. **(c)** Inverse relation of paired-pulse ratio (PPR) and intrinsic Pr for various synaptic configurations. Dark purple arrows indicate the PPR corresponding to 80 VDCCs and light purple correspond to 100 VDCCs for canonical synapse. **(d)** Amount of Ca^{2+} ions bound to SERCA in response to a paired-pulse. **(e)** Variation in peak calcium concentration at the active zone, $[Ca^{2+}]_{AZ}$, with the number of VDCCs. **(f)** Cumulative calcium concentrations over 20 ms at the active zone in response to the second AP in a paired-pulse protocol for various Pr. **(g)** Calcium concentration at the active zone, $[Ca^{2+}]_{AZ}$ for Pr = 0.1. The colored text describes the corresponding number of VDCCs in each of the configurations to arrive at Pr = 0.1. Inset: Box area zoomed-in to show details of base level $[Ca^{2+}]_{AZ}$ concentration after the second AP. **(h)** Variation of paired-pulse ratio for different ISI. Data are mean ± s.d.

Supplementary Figure 6: Response of the bouton to a stimulus of 20 pulses at 10 Hz in canonical synapse.

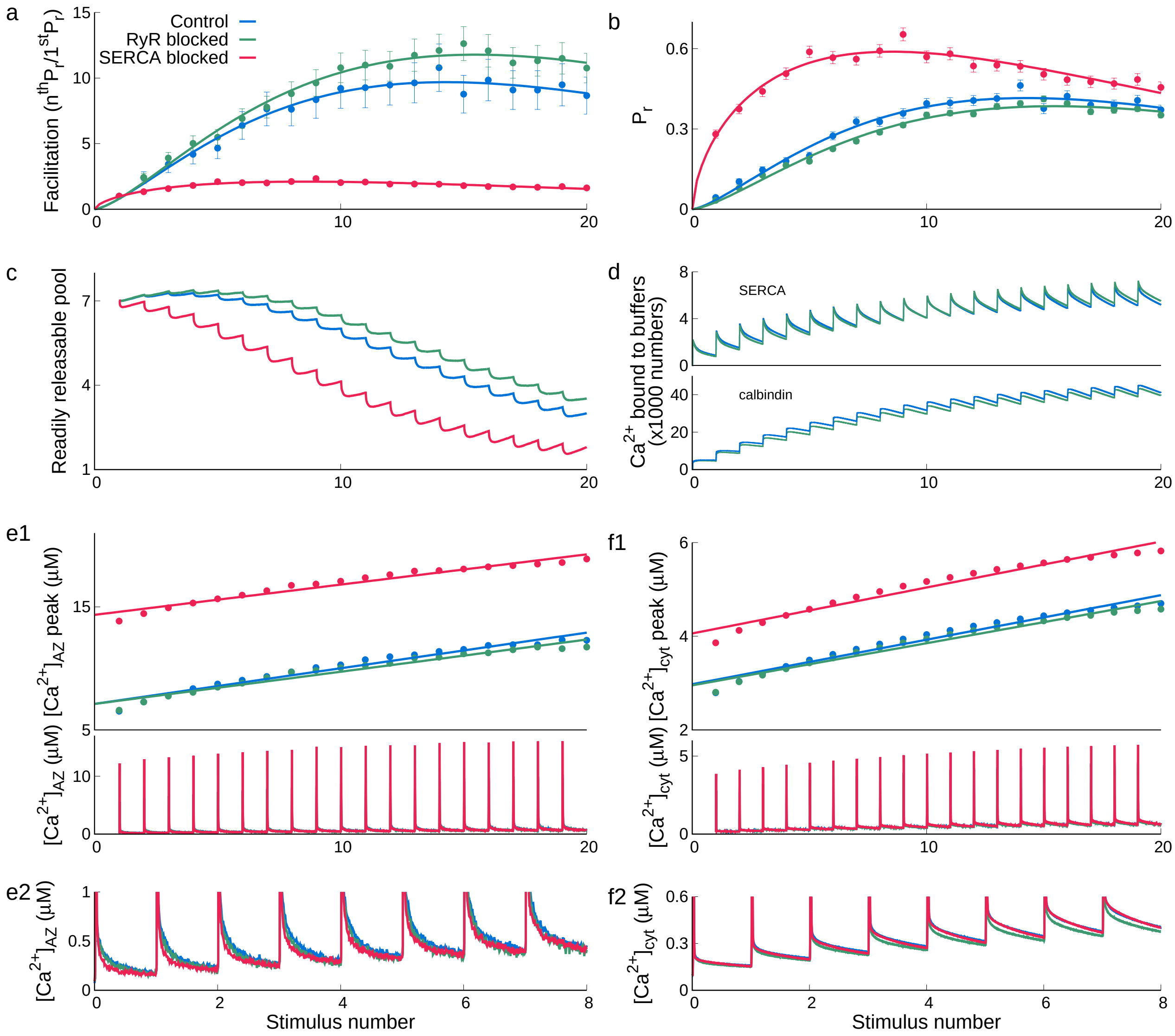

Figure 6. (a) Facilitation for a synapse with Stores blocked is lower than Control. **(b)** Release probability of a vesicle (Pr) for each AP in the train. **(c)** Decrease in the RRP due to successive releases. **(d)** Calcium ions binding to SERCA (top) and calbindin-d28k (bottom). **(e1)** Peak of calcium at the active zone, $[Ca^{2+}]_{AZ}$ (top) and trace of average calcium at the active zone (bottom), and **(e2)** zoom-in of bottom panel of (e1) showing the differences in basal calcium levels. **(f1)** Peak of calcium in the entire bouton, $[Ca^{2+}]_{cyt}$ (top) and trace of average calcium in the entire bouton (bottom), and **(f2)** zoom-in of the bottom panel of (f1) showing the differences in basal calcium levels. Data are mean $\pm$ s.d.

Supplementary Figure 7: Response of the bouton to a stimulus of 20 pulses at 50 Hz in canonical synapse.

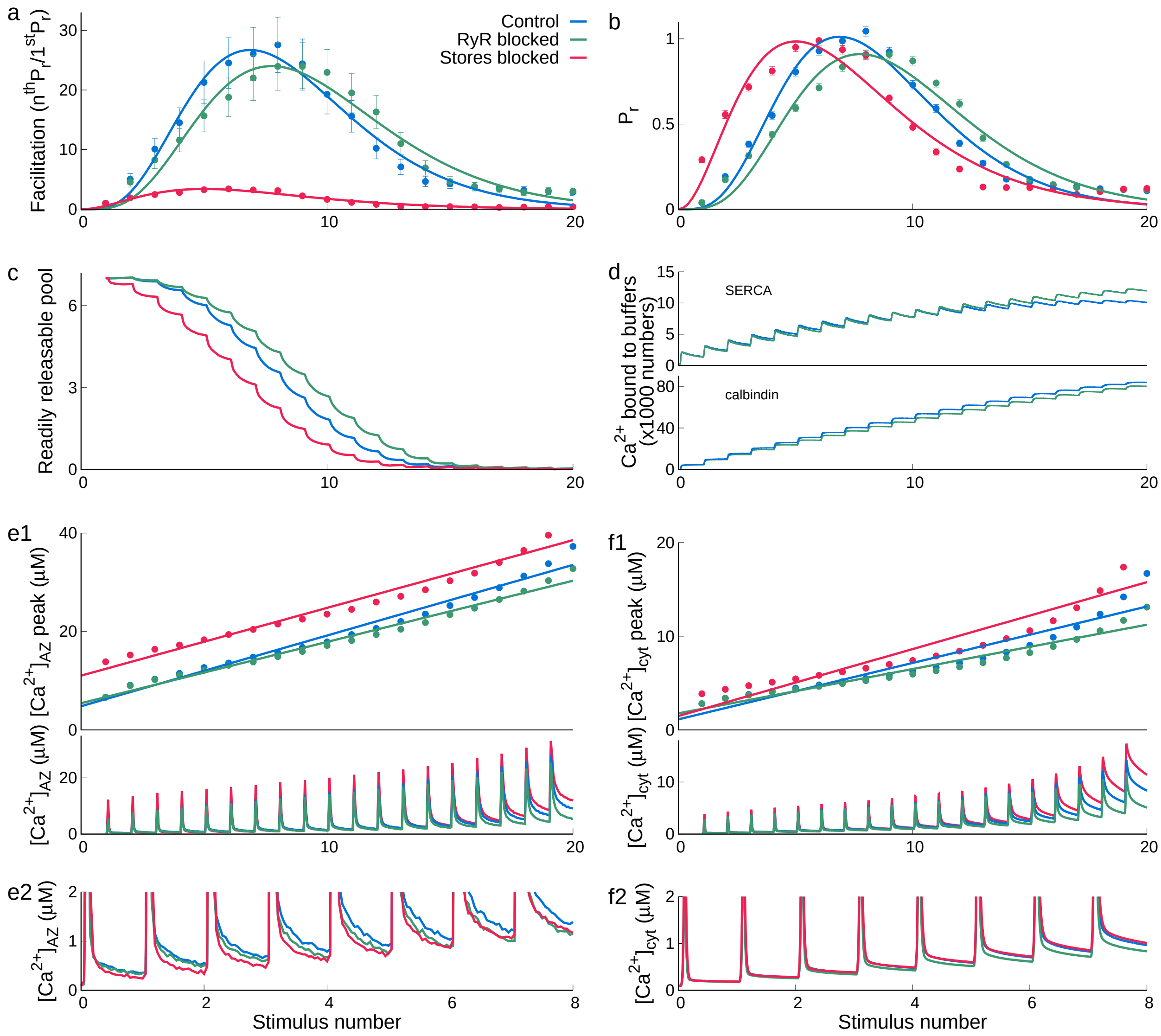

Figure 7. **(a)** Facilitation for a synapse with Stores blocked is lower than Control. **(b)** Release probability of a vesicle (Pr) for each AP in the train. **(c)** Decrease in the RRP due to successive releases. **(d)** Calcium ions binding to SERCA (top) and calbindin-d28k (bottom). **(e1)** Peak of calcium at the active zone, $[Ca^{2+}]_{AZ}$ (top) and trace of average calcium at the active zone (bottom), and **(e2)** zoom-in of bottom panel of (e1) showing the differences in basal calcium levels. **(f1)** Peak of calcium in the entire bouton, $[Ca^{2+}]_{cyt}$ (top) and trace of average calcium in the entire bouton (bottom), and **(f2)** zoom-in of the bottom panel of (f1) showing the differences in basal calcium levels. Data are mean $\pm$ s.d.

Supplementary Figure 8: Synchronous and asynchronous vesicle release for train stimulus.

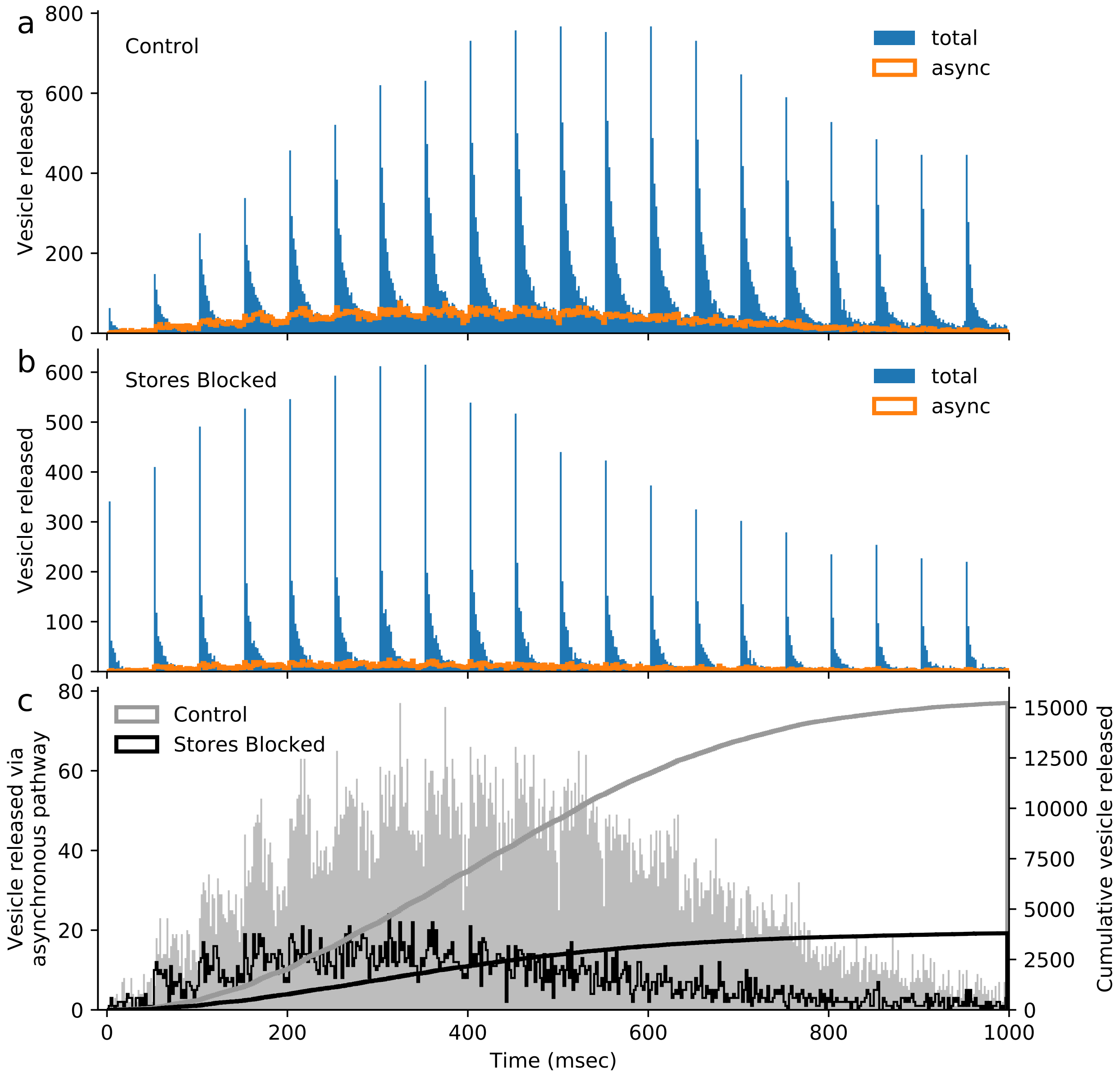

Figure 8. **(a)** Total vesicles released in 2000 trials of Control synapse. **(b)** Total vesicles released in 2000 trials of Stores blocked synapse. **(c)** Comparison of asynchronous release between the two synaptic configurations.

Supplementary Figure 9: RyR and IP3R play a minor role in facilitation.

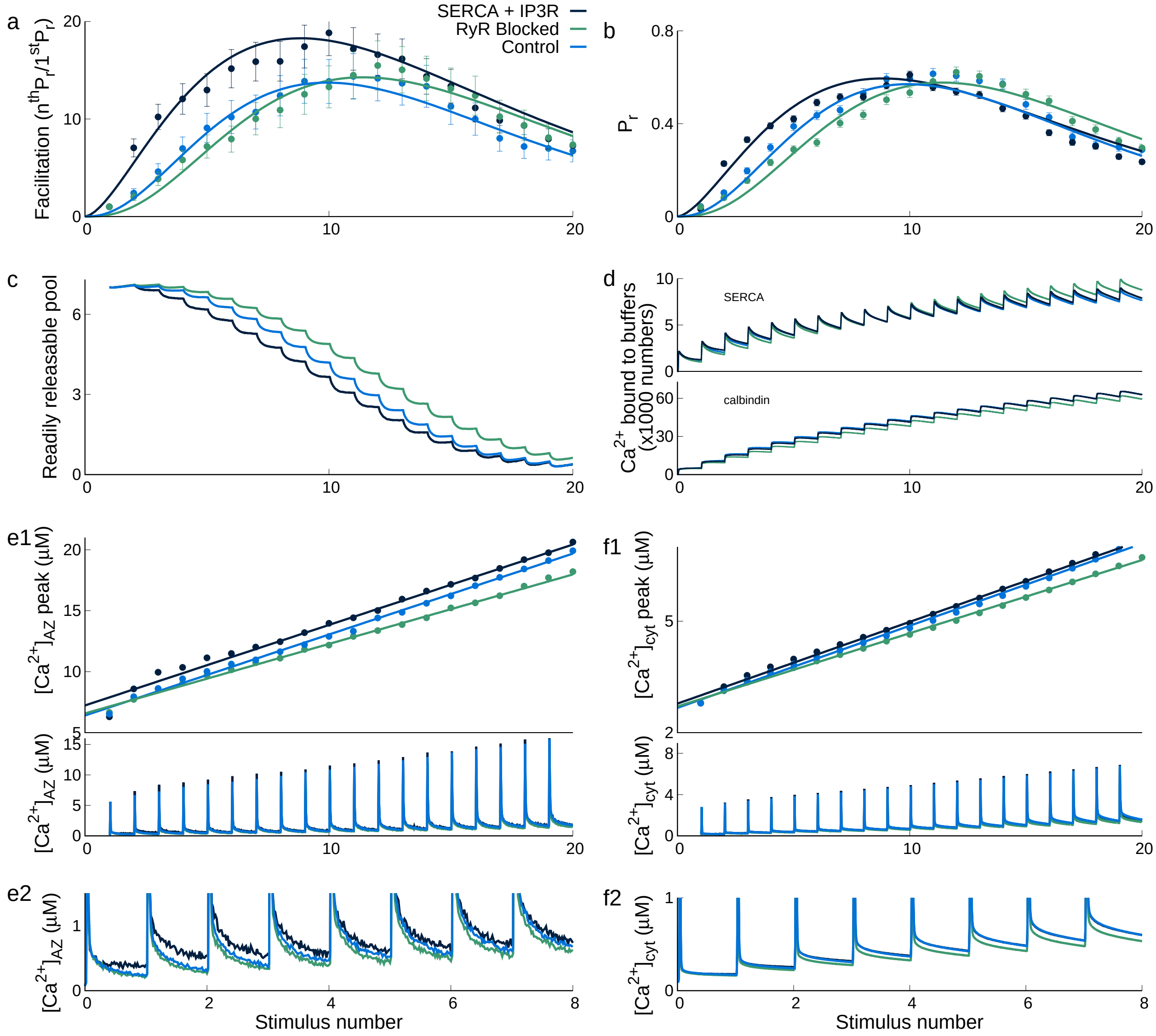

Figure 9. Ryanodine receptors have fast albeit low affinity calcium binding sites that are rapidly activated, however remain active only at high amplitudes of calcium[2]. On the other hand, IP3 receptors have slower but high affinity calcium binding sites for activation but also require IP3 to bind to the appropriate binding site on the receptor[3]. The slow inactivation rates and high affinity for calcium allow IP3Rs to sustain calcium release for several seconds after the VDCCs have closed. Owing to these biophysical properties, both IP3Rs and RyRs differentially contribute to facilitation in addition to SERCA. Data are mean ± s.d.

Supplementary Figure 10: Binomial release.

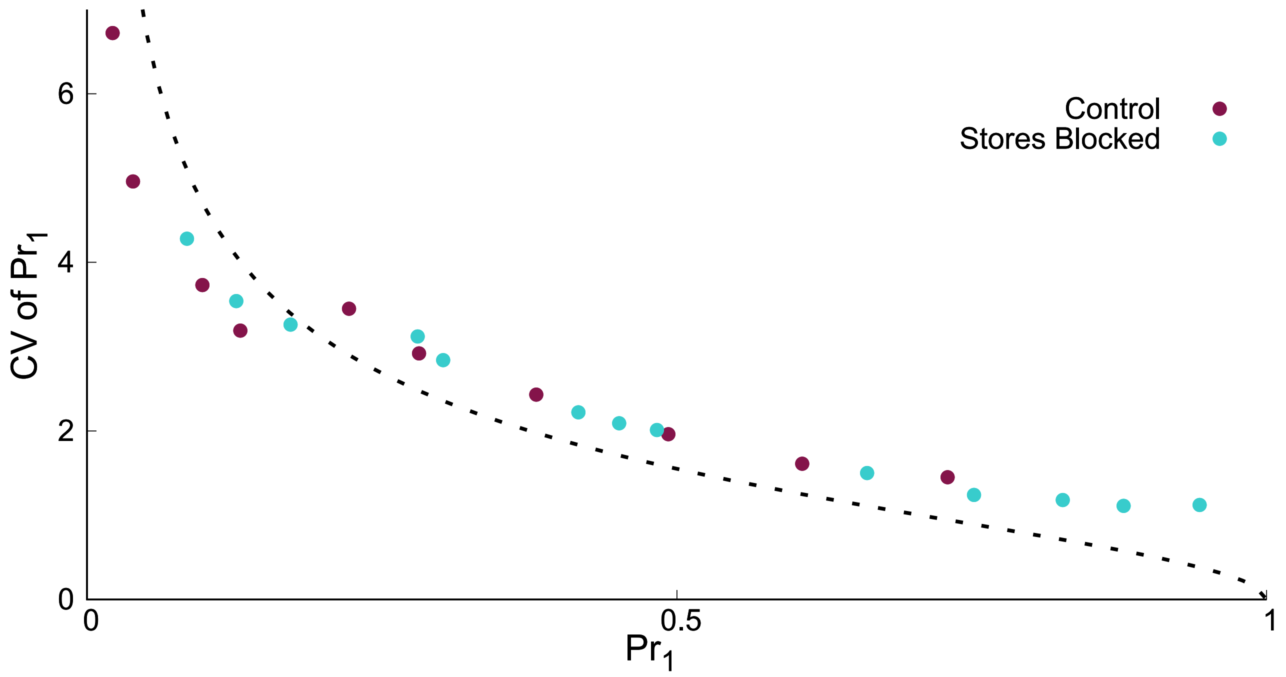

Figure 10. Measured coefficient of variation of Pr_1 for both Stores blocked and Control synapse configurations follows $CV = \sqrt{((1-p)/(np))}$, where p is release probability and n is number of active sites, shown with a dashed line.

Supplementary Figure 11: VDCC distribution.

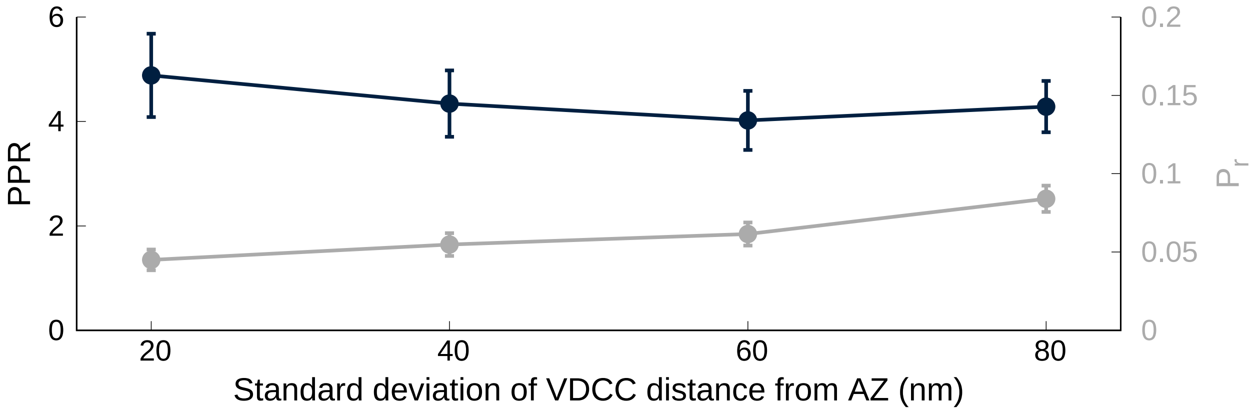

Figure 11. At a mean distance of 350 nm from active zone VDCCs were distributed with varying standard deviation. For each of the cases, we see that there is no significant change in Pr. Data are mean ± s.d.

Supplementary Figure 12: Calcium diffusion in presence of mobile and immobile buffers.

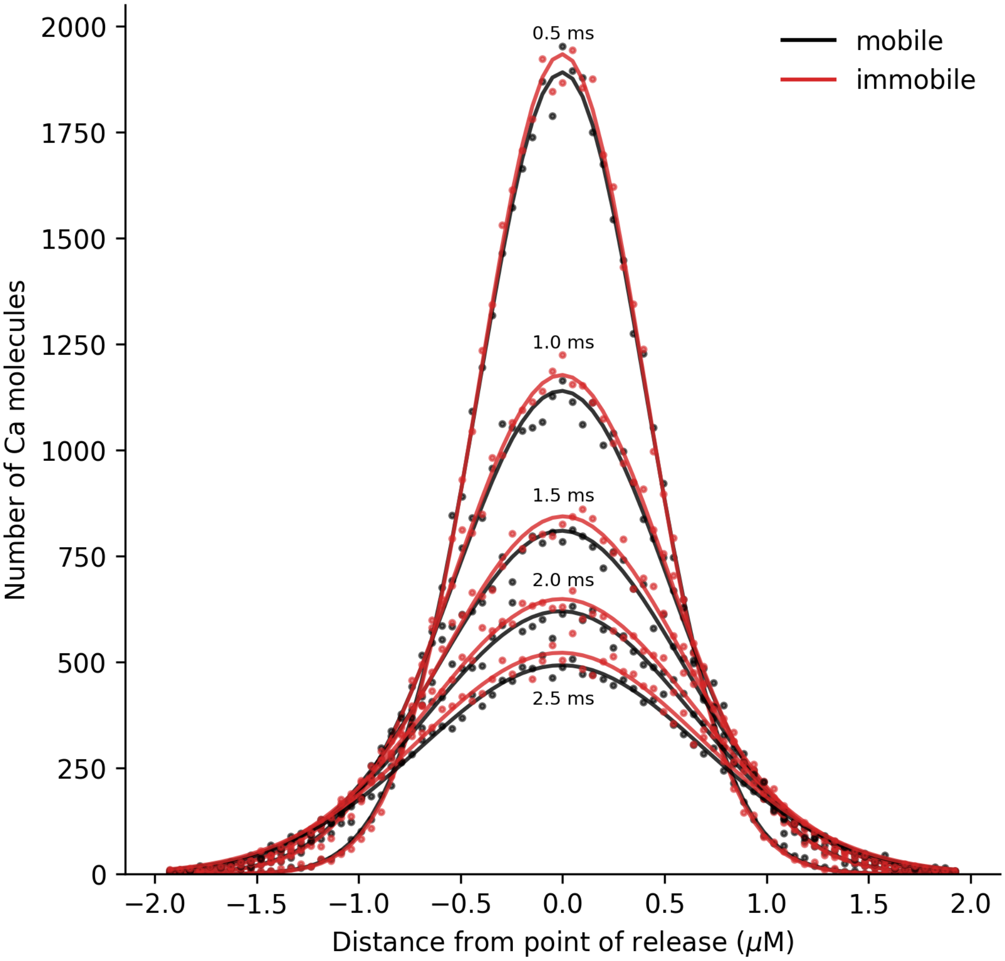

Figure 12. Data points are fitted with black line (mobile buffer) and red line (immobile buffer). We looked at the distribution of free calcium after it is released at a point in space, at different times. Five instances of calcium distribution are shown at an interval of 0.5 ms. We observe that calcium distribution profile is not modified by the mobility of buffers. The data is fitted with a gaussian function ($f(x) = Ae^{-x^2/b}$). Calbindin-D28k diffusion constant: $0.28 \times 10^{-6} cm^2 s^{-1}$.

Supplementary References

1. Venkatesh N Murthy, Terrence J Sejnowski, and Charles F Stevens. Heterogeneous release properties of visualized individual hippocampal synapses. *Neuron*, 18(4):599–612, 1997.

2. Elena Saftenku, Alan J Williams, and Rebecca Sitsapesan. Markovian models of low and high activity levels of cardiac ryanodine receptors. *Biophysical journal*, 80(6):2727–2741, 2001.

3. Gary W De Young and Joel Keizer. A single-pool inositol 1, 4, 5-trisphosphate-receptor-based model for agonist-stimulated oscillations in ca2+ concentration. *Proceedings of the National Academy of Sciences*, 89(20):9895–9899, 1992.
